# High-throughput iSpinach fluorescent aptamer-based real-time monitoring of in vitro transcription

**DOI:** 10.1186/s40643-022-00598-0

**Published:** 2022-10-27

**Authors:** Weitong Qin, Liang Li, Fan Yang, Siyuan Wang, Guang-Yu Yang

**Affiliations:** 1 State Key Laboratory of Microbial Metabolism, Joint International Research Laboratory of Metabolic and Developmental Sciences, School of Life Sciences and Biotechnology, Shanghai Jiao Tong University, Shanghai, 200240 China; 2 Hzymes Biotechnology Co. Ltd, Hubei, 430010 China

**Keywords:** In vitro transcription, RNA aptamer, T7 RNAP, Real-time detection

## Abstract

In vitro transcription (IVT) is an essential technique for RNA synthesis. Methods for the accurate and rapid screening of IVT conditions will facilitate RNA polymerase engineering, promoter optimization, and screening for new transcription inhibitor drugs. However, traditional polyacrylamide gel electrophoresis (PAGE) and high-performance liquid chromatography methods are labor intensive, time consuming and not compatible with real-time analysis. Here, we developed an inexpensive, high-throughput, and real-time detection method for the monitoring of in vitro RNA synthesis called iSpinach aptamer-based monitoring of Transcription Activity in Real-time (STAR). STAR has a detection speed at least 100 times faster than conventional PAGE method and provides comparable results in the analysis of in vitro RNA synthesis reactions. It also can be used as an easy and quantitative method to detect the catalytic activity of T7 RNA polymerase. To further demonstrate the utility of STAR, it was applied to optimize the initially transcribed region of the green fluorescent protein gene and the 3T4T variants demonstrated significantly enhanced transcription output, with at least 1.7-fold and 2.8-fold greater output than the wild-type DNA template and common transcription template, respectively. STAR may provide a valuable tool for many biotechnical applications related to the transcription process, which may pave the way for the development of better RNA-related enzymes and new drugs.

**Graphical Abstract:**

**Figure:**
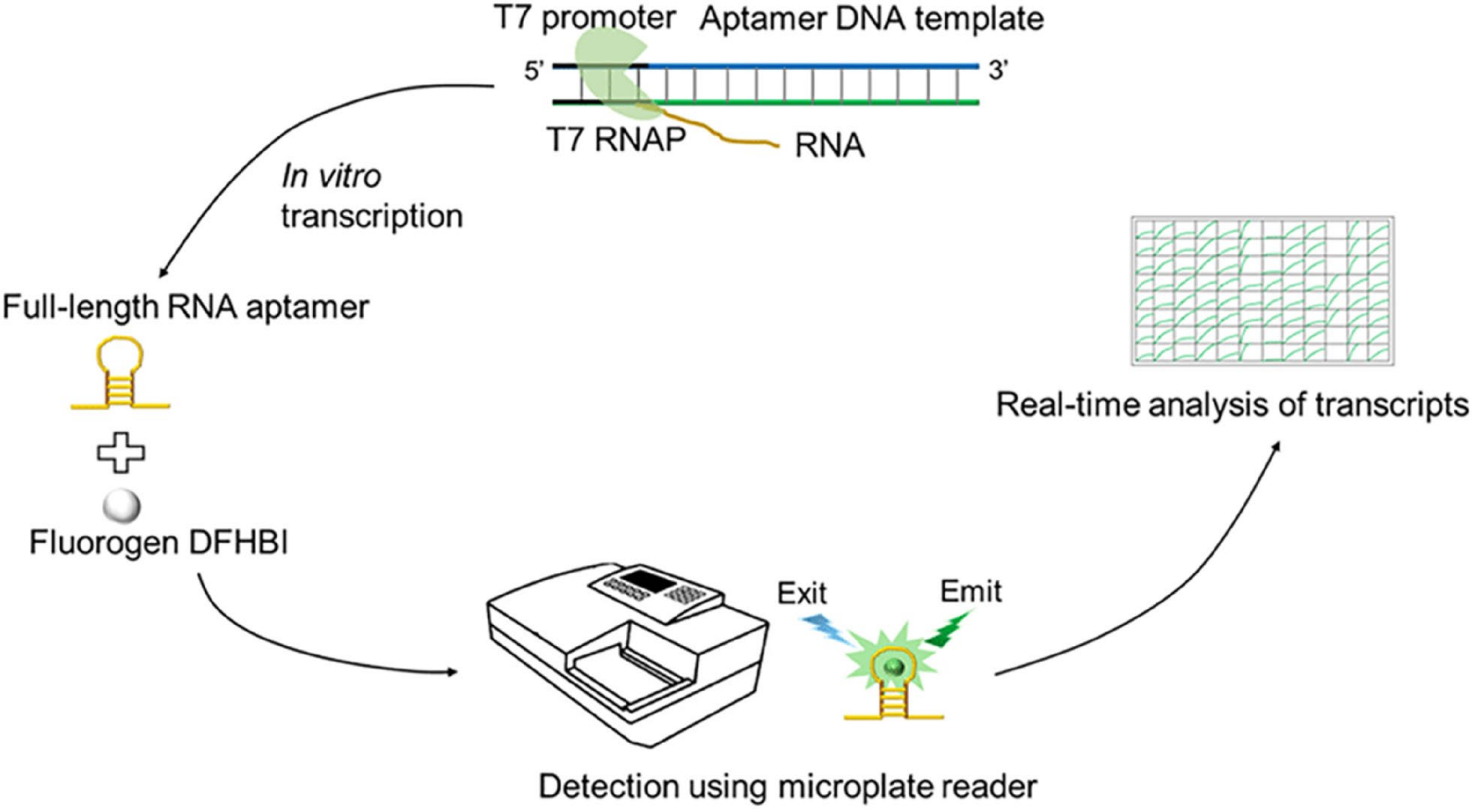
iSpinach aptamer-based monitoring of transcription activity in real-time (STAR).

**Supplementary Information:**

The online version contains supplementary material available at 10.1186/s40643-022-00598-0.

## Introduction

Transcription is among the most important biological reactions. In vitro transcription (IVT) is used to synthesize mRNA vaccines (Jain et al. 2021), screen RNA polymerases (RNAPs) (Chelliserrykattil and Ellington 2004), analyze promoter strength (Paul et al. 2013), and identify drugs that inhibit transcription (Villicana et al. 2014). However, the process of IVT will produce by-products, such as dsRNA and truncated fragments, which could reduce the quality and quantity of mRNA drug. Accordingly, it is of great importance to optimize the IVT system for both scientific community and pharmaceutical industry.

Gel electrophoresis remains the mainstream method for analyzing full-length RNA transcripts. The RNA yield and purity can only be analyzed using fluorescent dyes or radioisotopes after IVT is completed. Transcription by-products, such as abortive products (2–10 nucleotide) (Gong and Martin 2006), double-stranded RNA (Baiersdorfer et al. 2019), and truncated RNA products, typically produce smeared bands on polyacrylamide gel electrophoresis (PAGE). High-performance liquid chromatography and nucleic acid mass spectrometry are more accurate tools for quantitative RNA analysis (Burcar et al. 2013; Kanavarioti 2019), but are low-throughput and time- and labor-intensive.

Fluorescent light-up RNA aptamers (FLAPs) (Ouellet 2016) enable direct visualization and tracking of RNA molecules (Su and Hammond 2020). Various FLAPs have been developed that can form specific conformations by binding with fluorogen to generate fluorescence (Chen et al. 2019; Dao et al. 2021). Different aptamers have divergent folding properties and stabilities and are affected by the surrounding RNA conformation, reaction buffer, and other factors (Trachman et al. 2018). FLAPs have been widely used to measure gene transcription (Ying et al. 2019), observe intracellular RNA localization (Guzman-Zapata et al. 2017), and detect RNA aptamer binding to metabolites (Zheng et al. 2022). FLAPs are powerful tools in RNA research for observing RNA synthesis. Some in vitro RNA detection systems have been developed based on FLAPs. Hofer and colleagues fused the Spinach aptamer to an RNA of interest; this aptamer was cleaved by a hammerhead ribozyme after transcription, resulting in the generation of fluorescence (Hofer et al. 2013). However, this method has some variables, such as hammerhead ribozyme’s cleavage efficiency being easily affected by different buffers, existing unspecific cleavage because of its cleavage site being “GUX”, and the aptamer’s high salt ion dependence. These variables may lead to inconsistency between the fluorescent intensity and RNA yield. Kartje and colleagues utilized a hybrid DNA template containing a double-stranded promoter and downstream single-stranded Broccoli aptamer sequence (Kartje et al. 2021); the fully double-stranded DNA template was used in IVT, which has limited applications. Although these methods can be used to directly observe IVT, both methods suffer from low folding efficiency and poor thermostability because the aptamers are not suitable for use in vitro. Therefore, establishing a rapid, simple and less affected by external environment method will provide a valuable tool for many biotechnical applications using IVT.

In this study, we established a high-throughput, simple, and sensitive system for monitoring and quantifying RNA synthesis, named as iSpinach aptamer-based monitoring of transcription activity in real-time (STAR). We optimized the reaction conditions including metal ions, ribonucleotide triphosphates, DNA template, pH, and reaction temperatures. The activity of T7 RNAP was determined using STAR. To further demonstrate the utility of STAR, we optimized the 5′-untranslated region (UTR) sequence from + 1 to + 8 of the green fluorescent protein (GFP) gene. STAR can be used to easily and rapidly quantify T7 RNAP activity and optimize the 5′-UTR sequence, providing an efficient tool for various methods involving IVT.

## Methods

### DNA template used for STAR system

Antisense and sense single-stranded DNA templates containing the T7 promoter and a sequence encoding Spinach, tSpinach, Broccoli, tBroccoli, and iSpinach were synthesized by GenScript (Nanjing, China) (Additional file 1: Fig. S1). To prepare the double-stranded DNA template used in the STAR system, the antisense DNA template was annealed to the sense DNA template at a 1:1 molar ratio in diethylpyrocarbonate-treated water. Samples were heated to 95 °C for 5 min and then slowly cooled to 25 °C in a heating block for 30 min. The linear DNA template was obtained by polymerase chain reaction amplification of the other DNA templates used in the STAR system. The length of the double-stranded DNA was verified using agarose gel electrophoresis. The sequences are listed in Additional file 1: Table S1.

### Determination of the fluorescent intensity of different fluorescent RNA aptamer

The Spinach, tSpinach, Broccoli, tBroccoli, and iSpinach RNA transcripts were recovered and purified using a PAGE recovery kit (BioTeke, Beijing, China). The RNA product was eluted with RNase-free water. The purified RNA aptamers (1 μM) were unfolded by treatment at 85 °C for 5 min and then incubated at 25 °C for 20 min to promote their folding into a proper structure. The aptamers were mixed with 80 mM Tris–HCl pH 7.5, 2 mM MgCl_2_, 100 μM DFHBI, (MedChemExpress, Monmouth Junction, NJ, USA) and 200 mM K^+^ or Na^+^ in a 1:1 ratio. The mixtures were incubated at 25 °C for 15 min. The fluorescence intensity produced by different aptamers was determined using a microplate reader (SpectraMax M4, Sunnyvale, CA, USA).

The IVT reaction catalyzed by T7 RNAP contained 200 mM HEPES, pH 7.5, 30 mM MgCl_2_, 5 mM NaCl, 20 mM dithiothreitol, 0.2 U/μL murine RNase inhibitor (Hzymes, Hubei, China), 5 mM ribonucleotide triphosphate (NTP) mix (BBI Life Sciences, Shanghai, China), 0.002 U inorganic pyrophosphatase (Thermo Fisher Scientific, Waltham, MA, USA), 40 U T7 RNAP, and 100 μM DFHBI. The DNA templates of different RNA aptamers (20 nM) were added to the IVT reaction and then incubated at 37 °C for 20 min. The reaction was quickly placed on ice to stop the reaction. The fluorescence intensities produced by different aptamers were determined using a microplate reader (Ex: 469 nm, Em: 501 nm or Ex: 472 nm, Em: 507 nm) in a 96-well plate.

### Nucleic acid mass spectrometry

The obtained iSpinach RNA transcripts were purified using PAGE, the target band were cut from the PAGE gel, and the band were recovered and purified using a PAGE recovery kit (BioTeke). RNA was quantified at 50 pmol and analyzed using matrix-assisted laser desorption tandem time-of-flight mass spectrometry (MALDI TOF 7090, Shimadzu, Kyoto, Japan) and the data were analyzed using the MALDI Solutions software (Shimadzu).

### T7 RNAP enzyme expression and purification

A plasmid expressing T7 RNAP (pQE-80L) was transformed into *Escherichia coli* BL21 (DE3) cells (Transgene, Beijing, China). The cells were cultured in 1 L LB medium supplemented with ampicillin (50 μg/mL) at 37 °C to an OD_600_ of 0.6–0.8, after which expression was induced with 1 mM isopropyl β-D-1-thiogalactosidase at 37 °C for an additional 6 h. The cells were pelleted, treated with Buffer A (50 mM Tris–HCl, pH 8.0, 300 mM NaCl, 100 μM EDTA-Na^+^, and 3 mM imidazole), and lysed by high-pressure homogenization. The obtained lysate supernatant was centrifuged at 12,000 × *g* for 30 min. The lysate was purified using nickel-affinity chromatography. The resin was washed with five-bed volumes of Buffer A and ten-bed volumes of Buffer B (50 mM Tris–HCl, pH 8.0; 300 mM NaCl; 10% glycerin; 100 μM EDTA; and 10 mM imidazole) and eluted with five-bed volumes of Buffer C (50 mM Tris–HCl pH 8.0, 100 mM NaCl, 100 μM EDTA-Na^+^, 10% glycerin, and 300 mM imidazole). The purified protein was concentrated through an ultrafiltration tube (30 kDa; Millipore, Billerica, MA, USA) and exchanged with buffer D (20 mM Tris–HCl, pH 8.0, 100 mM NaCl, 100 μM EDTA-Na^+^) for three times. The protein concentration was determined by measuring the UV absorbance at 280 nm. The purified T7 RNAP was diluted to a final concentration of 5 mg/mL in storage buffer (50 mM Tris–HCl, pH 8.0; 100 mM NaCl; 100 μM EDTA-Na^+^; 1 mM dithiothreitol; and 75% glycerin), aliquoted, and stored at −80 °C.

### Real-time fluorescence measurement of IVT

The sample used for standard in vitro T7 RNAP transcription contained 200 mM HEPES, pH 7.5, 30 mM MgCl_2_, 5 mM NaCl, 20 mM dithiothreitol, 0.2 U/μL murine RNase inhibitor, 5 mM NTPs mix, 0.002 U inorganic pyrophosphatase, 120 nM DNA template, 40 U T7 RNAP, and 100 μM DFHBI. The reaction temperature was typically 37 °C. All reactions were performed in 96-well microtiter plates, and the relative fluorescence units (RFU) generated during IVT were measured in real-time using a microplate reader. Data were processed using Origin 10 software (OriginLab, Northampton, MA, USA).

### Denaturing PAGE of IVT products

After the IVT reaction was complete, the samples were treated with 1 U DNase I for 15 min at 37 °C to digest the transcription DNA template. EDTA was added at a final concentration of 60 mM to stop the reaction. The RNA transcripts were mixed with 2 × RNA loading dye (New England Biolabs, Ipswich, MA, USA) in a 1:1 ratio, boiled at 85 °C for 5 min, and immediately placed on ice to stop the reaction. The mixed sample (2 μL) was added to 15% urea-TBE denaturing polyacrylamide gel. The RNA transcript bands were stained with Gel-red dye (Solarbio, Beijing, China) for 5 min. A gel imaging system (Clinx, Shanghai, China) was used to observe the transcription band and acquire photos. The gray level of the gel band was measured using Quantity One software (Bio-Rad, Hercules, CA, USA).

## Results and discussion

### Screening fluorescent aptamers suitable for IVT

**Fig. 1:**
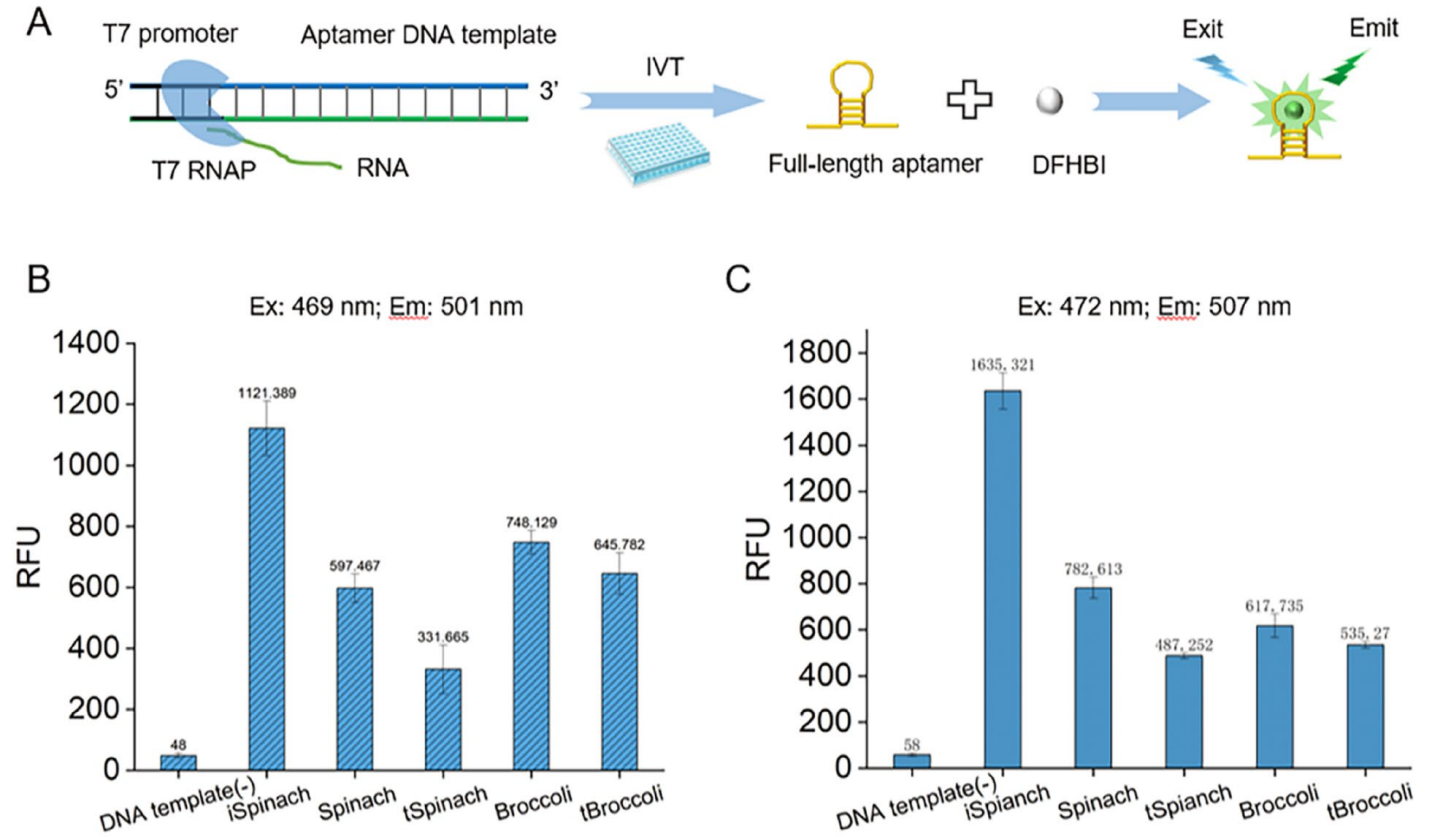
Screening a fluorescent aptamer suitable for in vitro transcription. **A** Schematic design of the real-time fluorescent assay method for monitoring the RNA synthesis. The DNA template contains a T7 promoter and downstream sequence encoding fluorescent RNA aptamer. **B** The relative fluorescence units (RFU) generated by five different RNA aptamers were measured under the condition that the excitation wavelength (Ex) was 469 nm and the emission wavelength (Em) was 501 nm. **C** The fluorescence intensities of five different RNA aptamers were measured under the condition that the Ex was 472 nm and the Em was 507 nm. Error bars represent the standard deviation of the triplicate experiments, P < 0.02

The RNA product generated from the IVT reaction does not generate fluorescence. To develop a simple, fast, low-cost, and real-time detection method for monitoring RNA synthesis, a double-stranded DNA template for IVT was designed to contain a common T7 promoter (TAATACGACTCACTATA), with a downstream sequence encoding a fluorescent RNA aptamer (Fig. 1a). Several fluorescent RNA aptamers have been reported but their fluorescence intensity, thermal stability, and metal ion dependence vary widely (Ouellet, 2016). To screen the most suitable RNA aptamer for real-time fluorescence monitoring in vitro, we selected five candidate fluorescent aptamers, Spinach (Paige et al. 2011), tSpinach, Broccoli (Filonov et al. 2014), tBroccoli, and iSpinach (Autour et al. 2016), and measured their RFU. Aptamer synthesis and fluorescence were monitored in real-time with a microplate reader in 50-μL reactions in a 96-well plate. Only full-length products folded into the proper structure and bound to the fluorogen DFHBI to produce a fluorescence signal, whereas the double-stranded RNA and abortive products did not generate fluorescence.

These aptamers were transcribed at 37 °C, and their RFU were measured at 25 °C in the presence of 100 mM metal ions (K^+^ or Na^+^), as some aptamers strongly depend on the presence of salt to fold correctly (Filonov et al. 2014). The results showed that iSpinach had relatively strong fluorescence at excitation wavelengths of 469 and 472 nm (Fig. 1b, c) and can function in a salt-free environment (Additional file 1: Fig. S2), indicating that iSpinach was suitable for the IVT reaction at 37 °C without K^+^ supplementation. Because of their low fluorescence in vitro, Broccoli, Spinach, tBroccoli, and tSpinach were not further evaluated in this study.

**Fig. 2:**
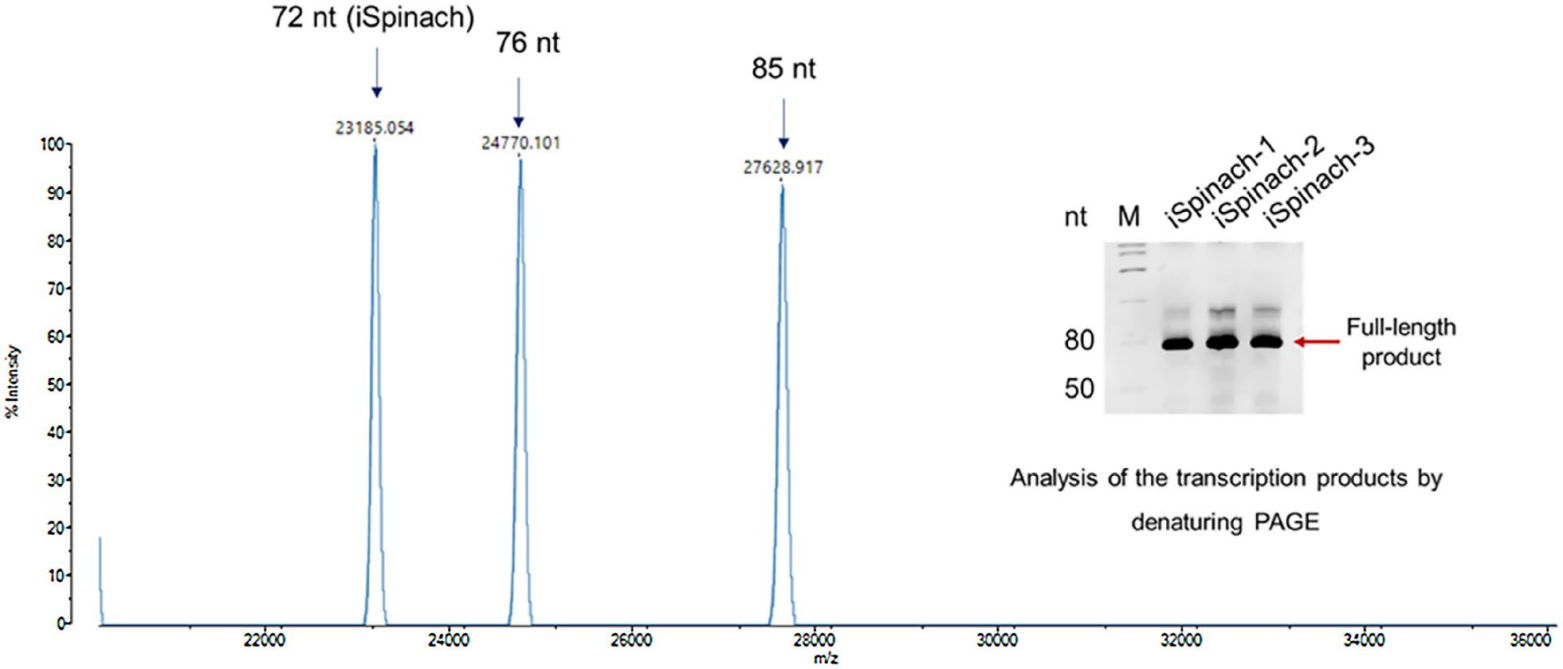
Analysis of iSpinach transcripts by denaturing polyacrylamide gel electrophoresis and nucleic acid mass spectrometry. M represents RNA marker. iSpinach-1, iSpinach-2, and iSpinach-3 represent three different replicates

To confirm this result, we analyzed the RNA transcripts using denaturing PAGE and nucleic acid mass spectrometry (Fig. 2). We observed the target iSpinach band (72 nucleotides), but there were also two other 3′-extended RNA products (76 and 85 nucleotides), which were longer than the full-length product. This result was expected, as T7 RNAP has weak RNA-dependent RNAP activity (Gholamalipour et al. 2018). The 3’-extended RNA products will generate fluorescence in the present of DFHBI. Nevertheless, we found this kind of byproduct usually is not exceed 10% of the total RNA output, so the impact on the results should be quite limited (Fernandez-Millan et al. 2017).

### Effects of ribonucleotide triphosphates and MgCl_2_ concentrations on the STAR system

To determine the best conditions for the STAR system, we optimized the concentrations of NTPs, MgCl_2_, monovalent ions, T7 RNAP; temperature; and pH. Beginning with our standard conditions, we determined the optimal concentration of iSpinach DNA template. The transcription yield reached a plateau when the concentration of DNA template exceeded 120 nM (Additional file 1: Fig. S3).

**Fig. 3:**
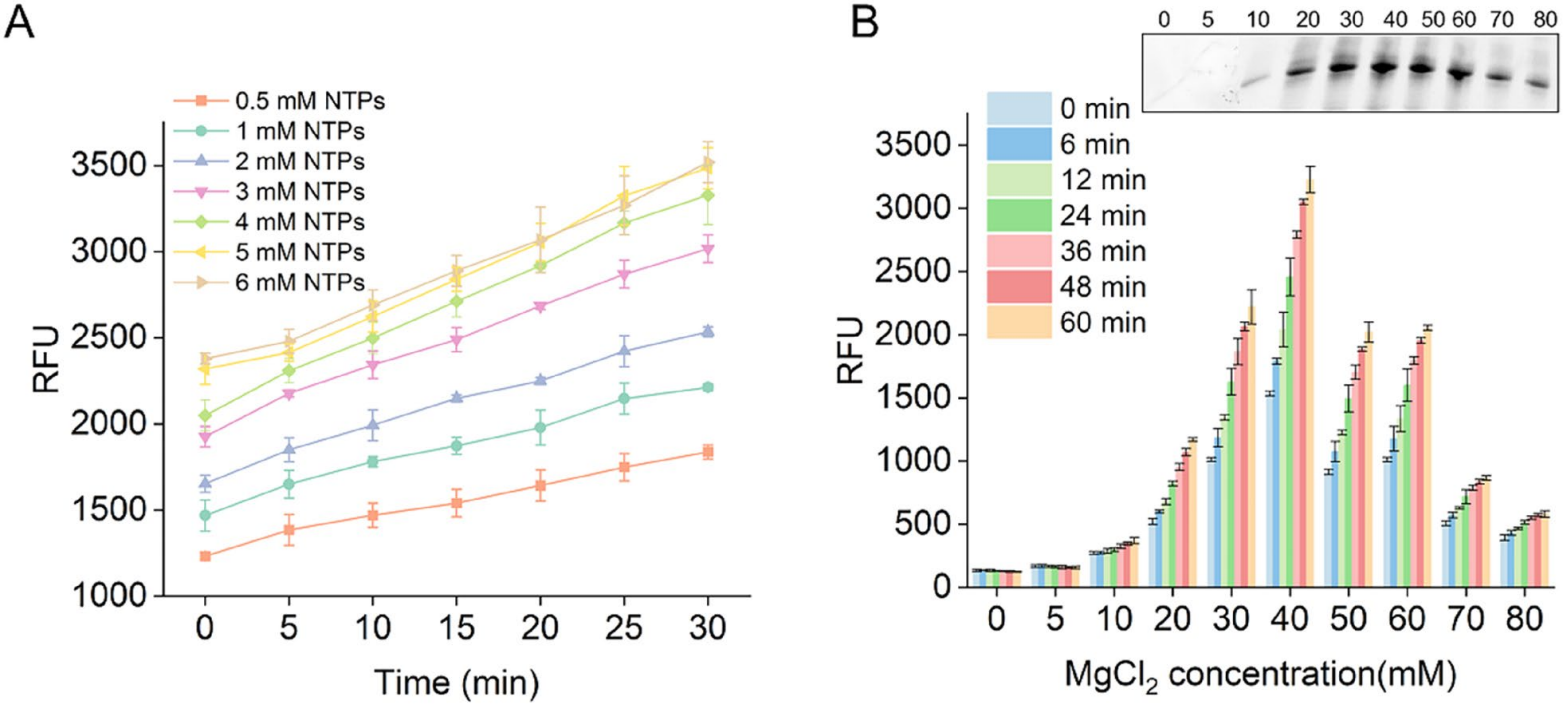
Effects of ribonucleotide triphosphate and MgCl_2_ concentrations on the STAR system. **A** Effect of NTP concentrations ranging from 0.5 mM to 6 mM. The relative fluorescent units were measured every 5 min. Error bars are standard errors of the mean (SEM). **B** Effect of MgCl_2_ concentrations ranging from 5 to 80 mM. The relative fluorescent units were measured every 6 min. The inset shows the results of denaturing PAGE analysis of the 60-min transcription products under different MgCl_2_ concentrations. Error bars represent the standard deviation of the triplicate experiments

NTPs (Liu et al. 2020) and MgCl_2_ (Yin and Steitz 2004) affect the efficiency of in vitro T7 RNAP transcription. Mg^2+^ has important roles in nucleotide addition cycles, and NTPs are substrates of IVT that directly chelate withMg^2+^. To explore the optimum conditions for the STAR system, we measured the RFU generated at NTP concentrations of 0.5–6 mM in real time (Fig. 3a). The results showed that the RFU, which represents the RNA yield, increased linearly over time at all seven NTP concentrations tested. The RNA yields also increased when the NTP concentrations ranged from 0.5 to 5 mM. However, when NTP concentration exceeded 5 mM, the RNA yield reached a plateau, indicating that 5 mM NTPs support greater RNA synthesis.

Next, we determined the effect of MgCl_2_ concentrations ranging from 5 to 80 mM on the STAR system under the optimum NTP concentration (Fig. 3b). We found that both limiting and excessive MgCl_2_ inhibited the activity of T7 RNAP; 40 mM MgCl_2_ was the optimal concentration for the STAR system. The RNA transcripts were also analyzed using denaturing PAGE. The RNA yield increased with increasing MgCl_2_ concentrations and was reduced at MgCl_2_ concentrations exceeding 40 mM, which is consistent with the fluorescence assay results.

### Effect of temperature, pH, and monovalent ions on the STAR system

**Fig. 4:**
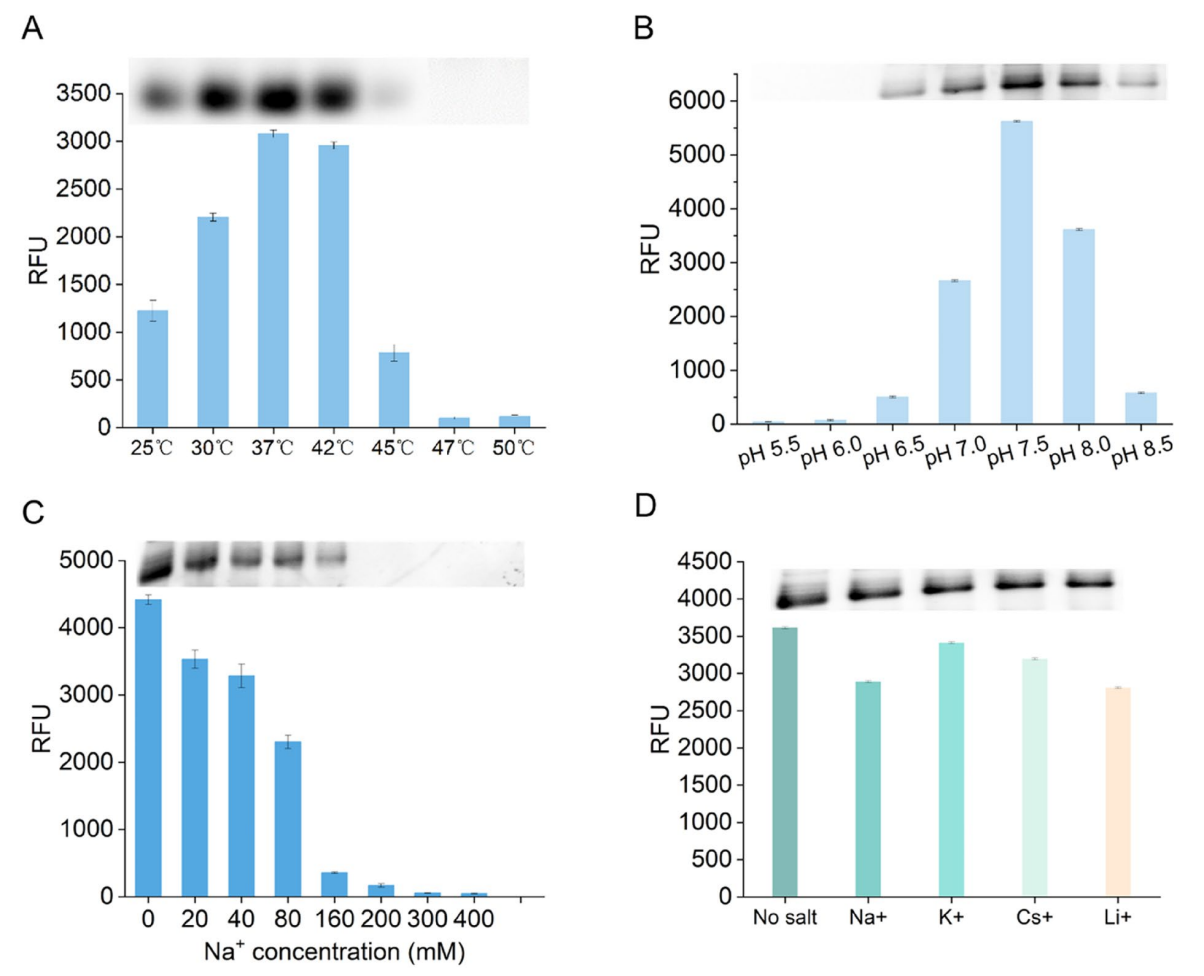
Effect of temperature, pH, and monovalent ions on the STAR system. **A** Relative fluorescence unit at a range of temperatures, the inset shows the results of denaturing polyacrylamide gel electrophoresis analysis of the 120-min transcription products. **B** Relative fluorescence unit for a range of pH values. **C** Relative fluorescence unit for a range of NaCl concentrations. **D** Relative fluorescence unit at 20 mM of different kinds of monovalent ions. Error bars represent the standard deviation of the triplicate experiments

Temperature is a key factor affecting the efficiency of IVT and affinity of iSpinach binding to DFHBI (Autour et al. 2016). To identify the optimum temperature for the STAR system, we performed transcription experiments at temperatures of 25–50 °C. Fluorescence increased with increasing temperatures from 25 °C to 37 °C and then decreased from 42 °C to 50 °C (Fig. 4a), indicating that iSpinach can form a stable complex with DFHBI at 37 °C. Denaturing PAGE analysis supported these results. The yield of RNA transcripts increased gradually with increasing temperatures but decreased by 80% at 45 °C, with no RNA transcripts observed at temperatures above 45 °C (Fig. 4a). The optimum temperature was approximately 37 °C and 42 °C. These results are consistent with the transcriptional activity of T7 RNAP, whose activity was lost at above 45 °C, indicating that the STAR system can accurately reflect the transcriptional activity of T7 RNAP at different temperatures.

The pH affects the charged state of enzymes and substrates (Rodrigues et al. 2013), thereby affecting enzyme activity. We measured the relative fluorescence intensity of the transcription reaction at pH 5.5–8.5. We found that the STAR system was highly sensitive to pH, and the optimum pH value was approximately 7.5, which was our standard condition (Fig. 4b).

Previous studies showed that T7 RNAP is highly sensitive to the ionic strength of ions such as Na^+^ and Cl^−^ (Orlov et al. 2018). Analysis of the effects of 20–400 mM NaCl on the STAR system showed that the RNA yield was strongly inhibited by NaCl. The activity decreased above 20 mM, with only 8.3% remaining activity at 160 mM (Fig. 4c). This result indicates that the system did not require addition of NaCl.

Next, we tested the effects of other monovalent ions on the STAR system, specifically 20 mM potassium, cesium, and lithium. The results showed that the STAR system is not sensitive to any of these monovalent ions, and thus it is not necessary to add them to the IVT reaction (Fig. 4d).

### Application of STAR in detecting the activity of T7 RNAP

T7 RNAP has wide applications in in vitro synthesis systems, such as IVT, cell-free transcription/translation systems, and isothermal amplification systems (Ju et al. 2021; Zhang et al. 2018). The catalytic activity of T7 RNAP not only affects the final yield of RNA transcripts, but also affects the rate of RNA synthesis. Traditional methods for determining the activity of T7 RNAP typically adopt the isotope method, which is prone to radioactive contamination and has high operating costs, making it difficult to achieve high-throughput (Padmanabhan et al. 2020). Therefore, establishing a simple and efficient method for detecting T7 RNAP activity is helpful for achieving high-throughput and automated activity detection, which is an urgent challenge in research related to T7 RNAP. To demonstrate that STAR can detect the activity of T7 RNAP, we purified T7 RNAP protein and measured the relative fluorescence intensities at T7 RNAP concentrations of 0.78–50 μg/mL in real-time using STAR.

**Fig. 5:**
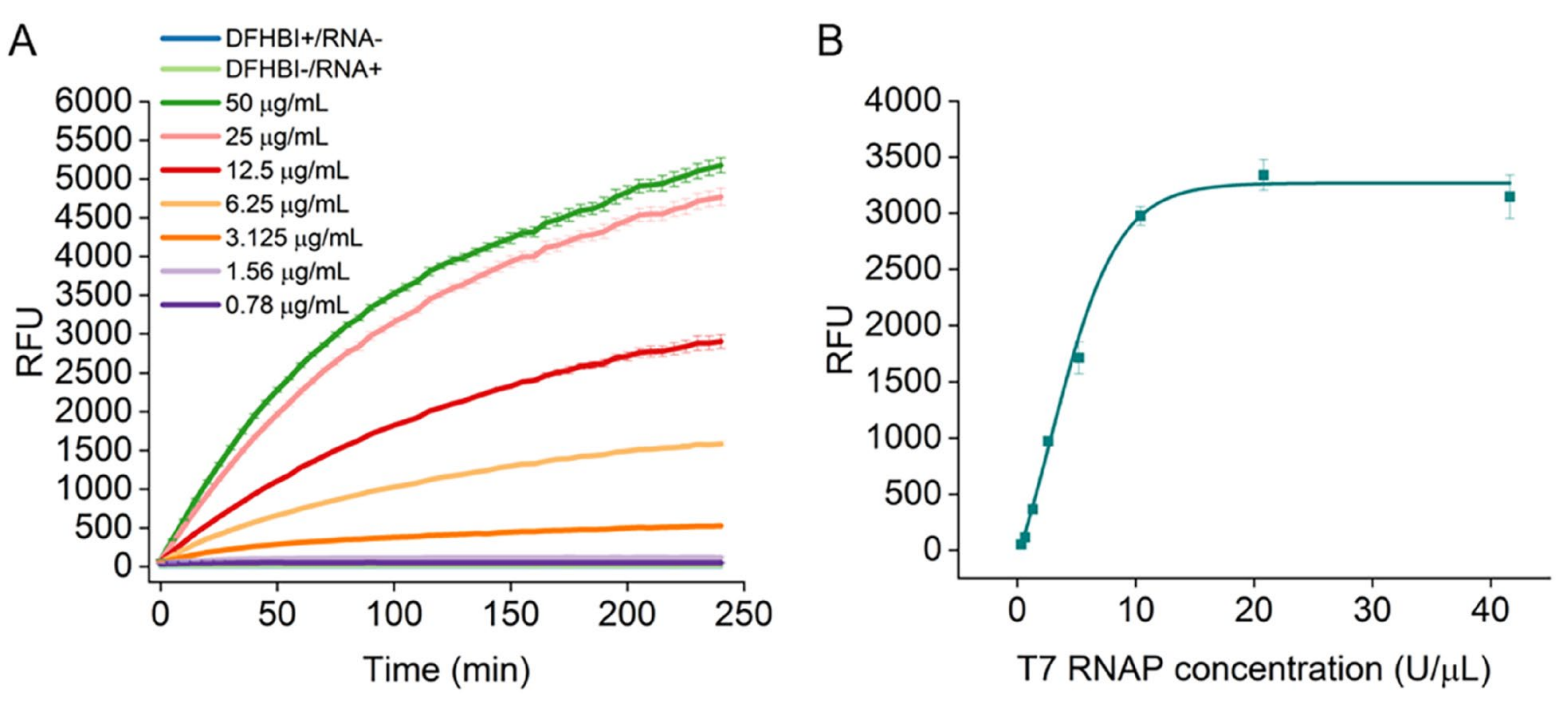
Application of STAR in characterizing the activity of T7 RNAP. **A** Real-time determination of relative fluorescence unit produced by STAR under different concentrations of T7 RNAP. **B** Real-time determination of relative fluorescence unit produced by STAR under different concentrations of commercial T7 RNAP. Error bars represent the standard deviation of the triplicate experiments

Our results showed that at a specific T7 RNAP concentration, the fluorescence intensity produced by STAR increased proportionally with time (Fig. 5a); the T7 RNAP concentration showed a linear relationship with the RNA transcripts produced by STAR from 0.75 to 25 μg/mL T7 RNAP (Fig. 5a). Nonetheless, beyond 25 μg/mL, T7 RNAP did not produce substantially improved yields (Fig. 5a). We also measured the fluorescence values generated by different concentrations of commercial T7 RNAP. The lowest concentration of T7 RNAP detected by STAR was 0.1 U/μL and the RNA yield reached a plateau once the concentration exceeded 10.4 U/μL. These results demonstrate that the STAR system accurately detected the activity of T7 RNAP within a certain concentration range. In addition, the T7 promoter of the DNA template can be replaced, and thus can be applied to characterize the activities of other types of polymerases such as SP6 RNAP and T3 RNAP.

### Application of STAR in optimizing the initially transcribed region of GFP gene

The complete transcription cycle catalyzed by T7 RNAP involves initiation, elongation, and termination steps (Steitz 2009). Initiation is the rate-limiting step of transcription. Some studies showed that the initially transcribed sequence (ITS) (8–10 base pairs) of DNA template strongly affect the stability of the initiation complex (Henderson et al. 2019). Inappropriate ITS can cause T7 RNAP to fall off the DNA template, resulting in the production of abortive products (2–10 nucleotides), thereby reducing the transcript yield. Therefore, designing a reasonable ITS can improve the production of full-length products and play an important role in RNA synthesis such as for mRNA vaccines. The ITS has been systematically mutagenized in previous studies and shown to affect transcription efficiency (Conrad et al. 2020; Imburgio et al. 2000). These previous studies provided a reliable reference for testing our STAR system.

To investigate the utility of STAR, the initially transcribed sequence (5′-UTR) from the + 1 to + 8 positions of the GFP gene was mutated with each mutation representing a single base-pair change to one of the three other base pairs. For example, the first three mutants contained A, C, or T in place of G at the first nucleotide of the 5′-UTR. To ensure that the measured fluorescence intensities were consistent with the levels of synthesized RNA, denaturing PAGE was performed following each transcription reaction.

**Fig. 6:**
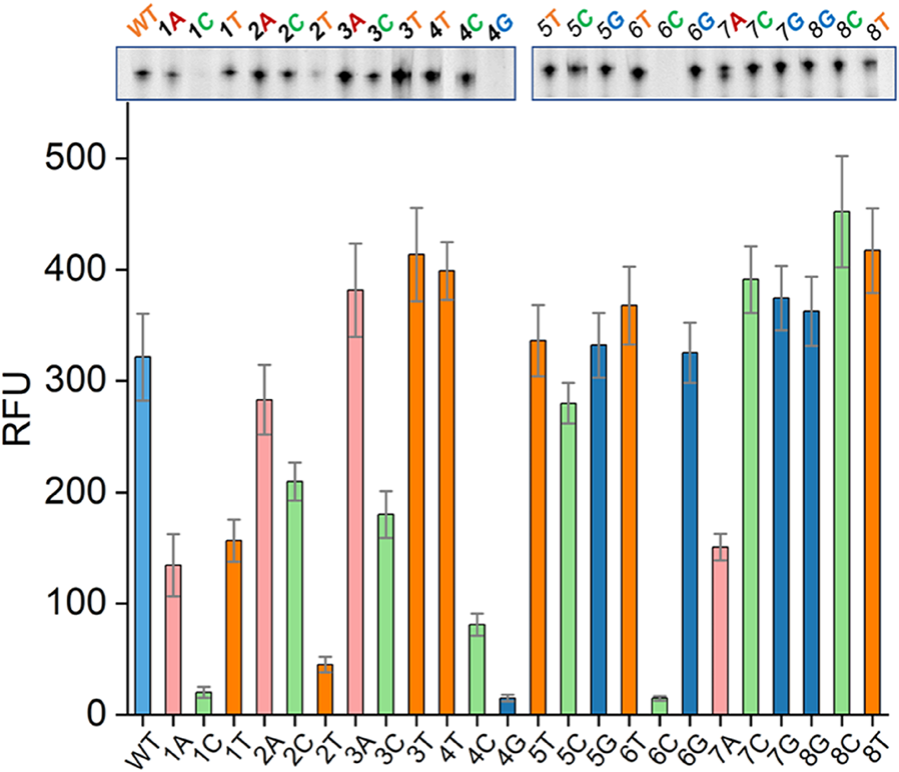
Transcription efficiency of systematically mutated initially transcribed regions of GFP gene quantified by STAR. The effect of substituting three other base pairs at each nucleotide position of 5′-UTR (from + 1 to + 8) in the DNA template was determined using STAR system. The *X*-axis represents different mutants, the *Y*-axis represents the relative fluorescence units. Error bars represent the standard deviation of the triplicate experiments, *P* < 0.05. The inset shows the results of denaturing polyacrylamide gel electrophoresis analysis of the 60-min transcription products

**Fig. 7:**
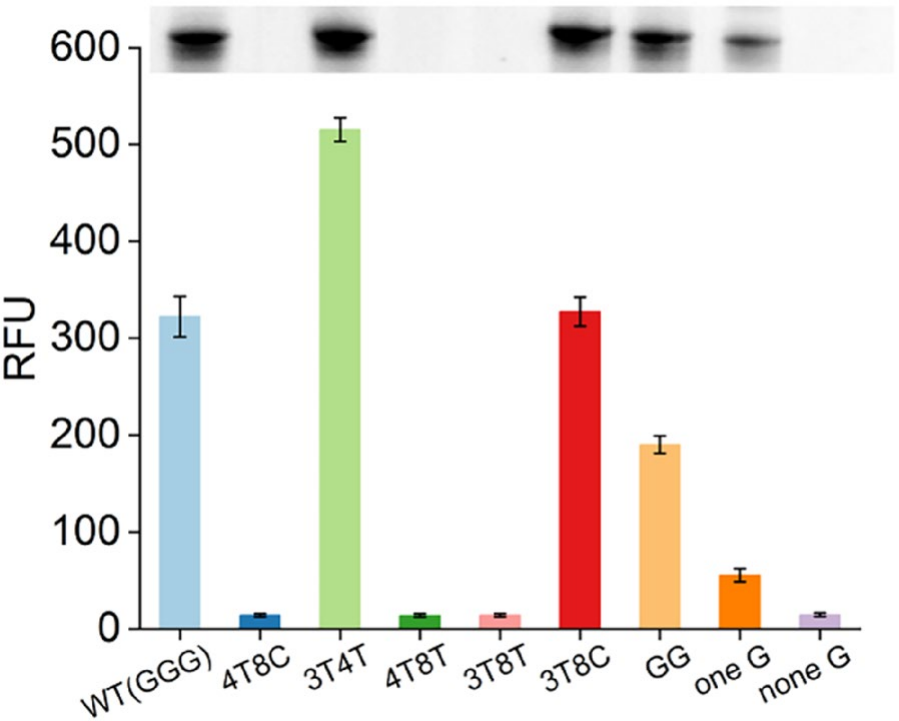
Transcription efficiency of combined mutants and truncated mutants quantified by STAR. The *X*-axis represents different mutants, the *Y*-axis represents the relative fluorescence units. Error bars represent the standard deviation of the triplicate experiments, *P* < 0.02. The inset shows the results of denaturing polyacrylamide gel electrophoresis analysis of the 60-min transcription products

We observed that transcripts with a G at position + 1 to + 2 were transcribed more efficiently than those with the three other mutants (Fig. 6). Substituting the G to A at position + 1 resulted in a ~ 93% loss in RNA yield, and the G to T mutant at position + 2 resulted in ~ 60% loss in RNA yield, which corresponded well with the PAGE results (Fig. 6). To confirm this result, we performed truncation experiments at positions + 1 to + 3; transcripts with a G triplet (WT-GGG) were transcribed more robustly compared to other truncated DNA templates containing two G, one G, or no G at positions + 1 to + 3 (Fig. 7). These results agree with previous findings showing that T7 RNAP depends on G at position + 1 to initiate transcription reactions and with common guidelines for T7 RNAP usage in RNA biology (Passalacqua et al. 2020).

The downstream sequences from + 4 to + 8 also affected transcription efficiency. Substitution of A-T or T-A base pairs with G-C or C-G base pairs at position + 4 resulted in ~ 62% or greater loss in the RNA yield (Fig. 6). Mutation of + 8A to C and T resulted in 36% and 25% increases in RNA yield, respectively (Fig. 6). However, substitution of + 6A with + 6C resulted in a ~ 100% loss in RNA yield (Fig. 6). These results indicate that this region is more prone to containing A-T or T-A base pairs, which facilitate the melting of DNA templates (Patwardhan et al. 2009).

Next, we combined the mutations in the DNA templates showing increased activity. The product transcribed from the 3T4T mutant was 50% higher than that of the wild type (WT-GGG), and the 3T8C mutant had little effect (Fig. 7). However, T7 RNAP did not recognize other combined mutations, such as 4T8C, 4T8T, and 3T8T (Fig. 7). These results indicate that the initially transcribed region strongly affects the transcriptional activity of T7 RNAP.

The target gene selected for transcription also leads to deviation in the transcription results (Conrad et al. 2020). For example, a mutation at position + 5 has little effect on the transcription efficiency of iSpinach, demonstrating that sequence of the initially transcribed region of different genes must be optimized. Nonetheless, our results generally agree with those of previous studies and suggest that the STAR system is reliable and can be used to rapidly optimize the 5′-UTR sequence. A traditional transcription reactions protocol to analyze RNA using PAGE method require gel preparation, sample loading, electrophoresis, and gel staining steps. The full process usually takes at least 180 min. The maximum throughput of one electrophoresis tank is often 60 samples. As a comparison, STAR method can monitor the transcription of 384 samples simultaneously. Only 5–10 min are required for the transcription of different samples and to make timely adjustments. Therefore, the detection rate of the STAR method is at least 100-fold faster than that of conventional PAGE.

## Conclusion

We developed a high-throughput, simple, and real-time iSpinach aptamer-based method for monitoring synthesized full-length RNA in vitro that can be easily implemented by most laboratories. This method is insensitive to metal ions, and no KCl is needed to give a strong fluorescent signal. And it can give more stable results than Höfer's method when targeting the same mRNA in the optimization of IVT system. We quantified the effects of the fundamental reaction components, reaction conditions, and initially transcribed region of the 5′-UTR sequence on real-time IVT. The NTP mix, MgCl_2_, and NaCl concentrations strongly influenced the total RNA yield, and most of our results supported and confirmed those of previous IVT studies using PAGE. The STAR system exhibited high thermostability, with an optimal reaction temperature between 37 °C and 42 °C, which is consistent with the conditions of conventional IVT reactions. In conclusion, this method only requires a conventional microplate reader and is at least 100-fold faster than traditional PAGE analysis. Our method can be used to rapidly optimize the IVT conditions, promoter strength, and 5′-UTR sequence and is useful for polymerase screening.

### Supplementary Information

**Additional file 1: Fig. S1.** Preparation of the DNA linear templates of different aptamers. M: Marker. 1, 2: Spinach DNA template. 3, 4: tSpinach. 5, 6: iSpinach. 7: Broccoli. 8: tBroccoli. **Fig. S2.** Effect of monovalent metal ions on the fluorescence of complexes formed by different aptamers with DFHBI. The reactions were performed at 25^o^C. **Fig. S3.** Effect of the DNA template concentrations on the STAR system. **Table S1.** Aptamer sequence used in this study. **Table S2.** Strains and plasmids used in this study. **Table S3.** Primers used in this study.

## Data Availability

The authors declare that all data supporting the findings of this study are available in the paper and its supplementary information files.

## Abbreviations

IVT: In vitro transcription
T7 RNAP: T7 RNA polymerase
FLAPs: Fluorescent light-up RNA aptamers
DFHBI: 3,5-Difluoro-4-hydroxybenzylidene imidazolinone
STAR: ISpinach aptamer-based monitoring of Transcription Activity in Real-time
RFU: Relative fluorescence units
PAGE: Polyacrylamide gel electrophoresis
NTP: Ribonucleotide triphosphate
ITS: Initially transcribed sequence
