# Supplementary material for: High-throughput iSpinach fluorescent aptamer-based real-time monitoring of in vitro transcription
Source: Bioresour Bioprocess. 2022 Oct 27;9(1):112. doi: 10.1186/s40643-022-00598-0 (PMC10991154; doi:10.1186/s40643-022-00598-0)
Supplement: Supplementary file 1 — Additional file 1: Fig. S1. Preparation of the DNA linear templates of different aptamers. M: Marker. 1, 2: Spinach DNA template. 3, 4: tSpinach. 5, 6: iSpinach. 7: Broccoli. 8: tBroccoli. Fig. S2. Effect of monovalent metal ions on the fluorescence of complexes formed by different aptamers with DFHBI. The reactions were performed at 25oC. Fig. S3. Effect of the DNA template concentrations on the STAR system. Table S1. Aptamer sequence used in this study. Table S2. Strains and plasmids used in this study. Table S3. Primers used in this study. [file 40643_2022_598_MOESM1_ESM.docx]

Additional file 1


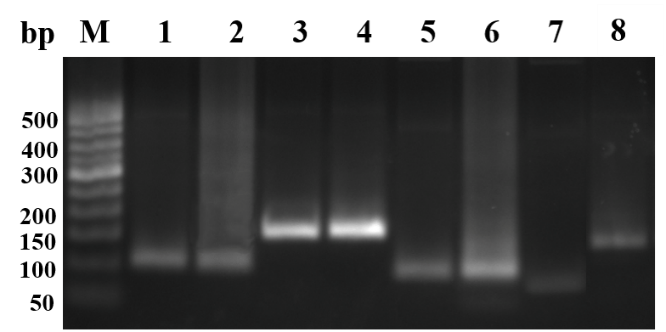


**Fig. S1** Preparation of the DNA linear templates of different aptamers. M: Marker. 1, 2: Spinach DNA template. 3, 4: tSpinach. 5, 6: iSpinach. 7: Broccoli. 8: tBroccoli.


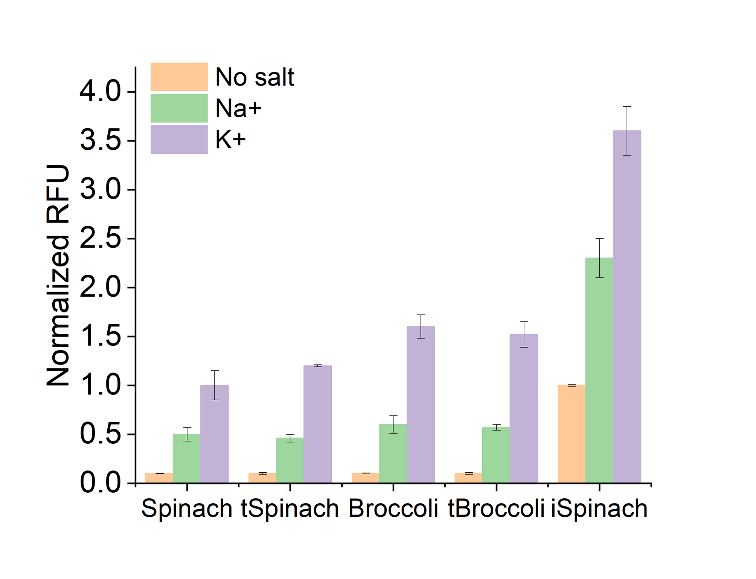


**Fig. S2** Effect of monovalent metal ions on the fluorescence of complexes formed by different aptamers with DFHBI. The reactions were performed at 25^o^C.


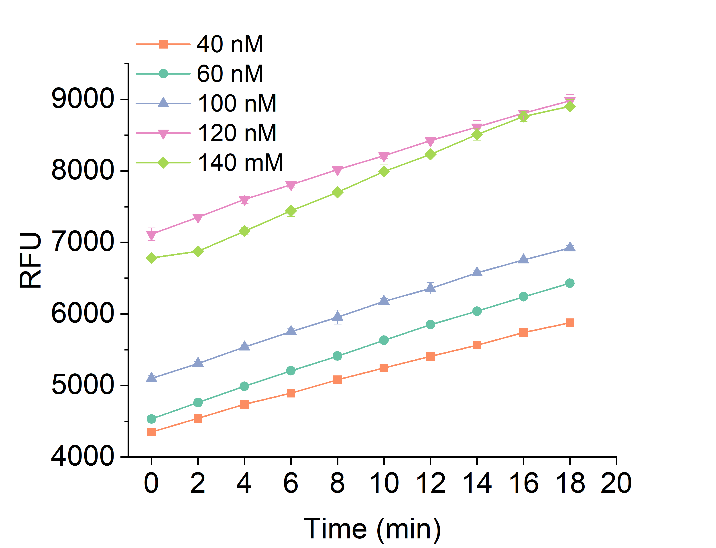


**Fig. S3** Effect of the DNA template concentrations on the STAR system.

**Table S1** Aptamer sequence used in this study.

| **Description** | **Sequence (5’-3’)** |
| --- | --- |
| Spinach | GTCAGATCCATAATACGACTCACTATAGGGGACGCGACCGAAATGGTGAAGGACGGGTCCAGTGCTTCGGCACTGTTGAGTAGAGTGTGAGCTCCGTAA CTGGTCGCGTC |
| tSpinach | GTCAGATCCATAATACGACTCACTATAGGGGCCCGGATAGCTCAGTCGGTAGAGCAGCGGCCGGACGCAACTGAATGAAATGGTGAAGGACGGGTCCAGGTGTGGCTGCTTCGGCAGTGCAGCTTGTTGAGTAGAGTGTGAGCTCCGTAACTAGTCGCGTCCGGCCGCGGGTCCAGGGTTCAAGTCCCTGTTCGGGCGCCA |
| Broccoli | GTCAGATCCATAATACGACTCACTATAGGGAGACGGTCGGGTCCAGATATTCGTATCTGTCGAGTAGAGTGTGGGCT |
| tBroccoli | GTCAGATCCATAATACGACTCACTATAGGGGCCCGGATAGCTCAGTCGGTAGAGCAGCGGAGACGGTCGGGTCCAGATATTCGTATCTGTCGAGTAGAGTGTGGGCTCCGCGGGTCCAGGGTTCAAGTCCCTGTTCGGGCGCCA |
| iSpinach | GTCAGATCCATAATACGACTCACTATAGGGGCGACTACGGTGAGGGTCGGGTCCAGTAGCTTCGGCTACTGTTGAGTAGAGTGTGGGCTCCGTAGT CGC |

**Table S2** Strains and plasmids used in this study

| **Name** | **Description** |
| --- | --- |
| *E.coli* DH5α | Plasmid Extraction |
| *E.coli* BL21(DE3) | Protein expression strain |
| pUC57-Broccoli | Linear DNA template preparation |
| pUC57-tBroccoli | Linear DNA template preparation |
| pUC57-Spinach | Linear DNA template preparation |
| pUC57-tSpinach | Linear DNA template preparation |
| pUC57-iSpinach | Linear DNA template preparation |

**Table S3** Primers used in this study

| **Primer name** | **Sequence (5’-3’)** |
| --- | --- |
| T7+Bro/Spi-F | GTCAGATCCATAATACGACTCA |
| T7+Bro-R | AGCCCACACTCTACTCGA |
| T7+tBro-R | GACGCGACCAGTTACGGAGCT |
| T7+Spi-R | TGGCGCCCGAACAGGGACTT |
| T7+tiSp-R | GCGACTACGGAGCCCACACTCTA |
| 5UTR-iSp-1G(WT) | AAGCTTTAATACGACTCACTATAGGGAAATAAGAGAGAGCGA |
| 5UTR-iSp-1A | AAGCTTTAATACGACTCACTATAAGGAAATAAGAGAGAGCGA |
| 5UTR-iSp-1C | AAGCTTTAATACGACTCACTATACGGAAATAAGAGAGAGCGA |
| 5UTR-iSp-1T | AAGCTTTAATACGACTCACTATATGGAAATAAGAGAGAGCGA |
| 5UTR-iSp-2A | AAGCTTTAATACGACTCACTATAGAGAAATAAGAGAGAGCGA |
| 5UTR-iSp-2C | AAGCTTTAATACGACTCACTATAGCGAAATAAGAGAGAGCGA |
| 5UTR-iSp-2T | AAGCTTTAATACGACTCACTATAGTGAAATAAGAGAGAGCGA |
| 5UTR-iSp-3A | AAGCTTTAATACGACTCACTATAGGAAAATAAGAGAGAGCGA |
| 5UTR-iSp-3C | AAGCTTTAATACGACTCACTATAGGCAAATAAGAGAGAGCGA |
| 5UTR-iSp-3T | AAGCTTTAATACGACTCACTATAGGTAAATAAGAGAGAGCGA |
| 5UTR-iSp-4T | AAGCTTTAATACGACTCACTATAGGGTAATAAGAGAGAGCGA |
| 5UTR-iSp-4C | AAGCTTTAATACGACTCACTATAGGGCAATAAGAGAGAGCGA |
| 5UTR-iSp-4G | AAGCTTTAATACGACTCACTATAGGGGAATAAGAGAGAGCGA |
| 5UTR-iSp-5T | AAGCTTTAATACGACTCACTATAGGGATATAAGAGAGAGCGA |
| 5UTR-iSp-5C | AAGCTTTAATACGACTCACTATAGGGACATAAGAGAGAGCGA |
| 5UTR-iSp-5G | AAGCTTTAATACGACTCACTATAGGGAGATAAGAGAGAGCGA |
| 5UTR-iSp-6T | AAGCTTTAATACGACTCACTATAGGGAATTAAGAGAGAGCGA |
| 5UTR-iSp-6C | AAGCTTTAATACGACTCACTATAGGGAACTAAGAGAGAGCGA |
| 5UTR-iSp-6G | AAGCTTTAATACGACTCACTATAGGGAAGTAAGAGAGAGCGA |
| 5UTR-iSp-7A | AAGCTTTAATACGACTCACTATAGGGAAAAAAGAGAGAGCGA |
| 5UTR-iSp-7C | AAGCTTTAATACGACTCACTATAGGGAAACAAGAGAGAGCGA |
| 5UTR-iSp-7G | AAGCTTTAATACGACTCACTATAGGGAAAGAAGAGAGAGCGA |
| 5UTR-iSp-8C | AAGCTTTAATACGACTCACTATAGGGAAATCAGAGAGAGCGA |
| 5UTR-iSp-8T | AAGCTTTAATACGACTCACTATAGGGAAATTAGAGAGAGCGA |
| 5UTR-iSp-8G | AAGCTTTAATACGACTCACTATAGGGAAATGAGAGAGAGCGA |
| 5UTR-3T4T | AAGCTTTAATACGACTCACTATAGGTTAATAAG |
| 5UTR-3T8C | AAGCTTTAATACGACTCACTATAGGTAAATCA |
| 5UTR-3T8T | AAGCTTTAATACGACTCACTATAGGTAAATTAG |
| 5UTR-4T8C | AAGCTTTAATACGACTCACTATAGGGTAATCA |
| 5UTR-4T8T | AAGCTTTAATACGACTCACTATAGGGTAATTA |
| 5UTR-two G | AAGCTTTAATACGACTCACTATAGGAAATAAG |
| 5UTR-one G | AAGCTTTAATACGACTCACTATAGAAATAAGA |
| 5UTR-none G | AAGCTTTAATACGACTCACTATAAAATAAGAGA |
| 5UTR-iSp-R | GGATCCGCGACTACGGAGCCCA |
